# The Development of a Collaborative Binational Strategy to Support the San Diego-Tijuana Transborder Community During the COVID-19 Pandemic

**DOI:** 10.3389/fpubh.2022.921513

**Published:** 2022-07-08

**Authors:** Barbara Jiménez, Justine Kozo

**Affiliations:** ^1^County of San Diego, Health and Human Services Agency, San Diego, CA, United States; ^2^Leaders Across Borders/Líderes a través de las Fronteras, San Diego, CA, United States; ^3^Leaders Across Borders/Líderes a través de las Fronteras, Tijuana, Mexico

**Keywords:** COVID-19 pandemic, COVID-19, border, transborder, cross-border collaboration, US-Mexico border, binational, health equity

## Abstract

The COVID-19 pandemic illustrates the need for and importance of cross-border public health collaboration. San Diego, California and Tijuana, Baja California are an interconnected region with one of the busiest international borders in the world and hundreds of thousands bi-directional crossings each day. As the sister cities witnessed the rising case numbers early in the pandemic, it became essential and urgent to implement a formal structure to facilitate cross-border COVID-19 communication, coordination, and collaboration. The present article describes how the development of a Collaborative Binational Strategy led to coordinated outreach and initiatives that addressed access and equity in the transborder region. Through examples, the article illustrates how regional leaders in San Diego and Tijuana harnessed existing transborder partnerships to collaboratively build infrastructure and communication pathways to exchange data, guidance, troubleshoot shared challenges, build capacity, and establish cross-border testing and vaccine opportunities. The challenges, lessons learned, and best practices may inform other multi-level, interdisciplinary, and cross-border jurisdictions on how to support a transborder community during a pandemic or other health emergency.

## Introduction

The start of the COVID-19 pandemic created similar challenges to residents and leaders in San Diego, California and Tijuana, Baja California; both experienced a rapid increase in cases, outbreaks, hospitalizations, and mortality. It was essential and urgent to leverage existing partnerships to develop a collaborative approach to address the needs of the transborder community. The County of San Diego Health and Human Services Agency engaged with its counterpart, the ISESALUD Tijuana Health Services Jurisdiction, and other key binational partners to establish a Binational Strategy to facilitate cross-border COVID-19 communication, coordination, and collaboration.

## Background and Rationale

The County of San Diego Health and Human Services Agency (HHSA) serves a diverse population of 3.3 million individuals. Thirty-four percent of the San Diego County population is Latino, there is a large immigrant community, and it is reported that over 450,000 (1) individuals speak a language other than English at home. San Diego is also home to an international border crossing, and it is believed to be one of the busiest in the world with over 150,000 individuals crossing the border northbound in a single day (2). People cross for many reasons, including work, recreation, shopping, education, family visitation, and healthcare access.

According to an estimate provided by the U.S. Consulate in Tijuana in an April 2020 email and data cited by the National Institute on Statistics and Geography (INEGI), ~132,000 to 265,000 U.S. citizens live in Baja California (3). There is also a large proportion of the San Diego essential workforce that live in Baja California and cross the border every day to work in agricultural fields, retail, hospitals, other healthcare facilities and many other critical industries. Many are on the front lines of the COVID-19 response.

While comprising a significant portion of the overall county population, the southern region of San Diego County is home to a large and growing Latino and transborder population (61.3% identify as Latino) (4) which has experienced the highest COVID-19 positivity rates in the county (5). Early in the pandemic, the Department of Homeland Security implemented border crossing restrictions, limiting trips for only essential reasons and for U.S. Citizens and Lawful Permanent residents only (6). When the restrictions were implemented in March 2020, crossings initially decreased to ~50,000 crossings per day, but then steadily increased over time to average ~100,000 per day by March 2021 (7). This highlights the transborder essential work force and region connectedness and reinforces the concern about potential disease transmission.

Within this context, the needs for cross-border communication and coordination were evident and garnered immediate support. The County of San Diego has established and maintained strong partnerships with counterparts in Baja California as part of the County's *Live Well San Diego* vision to achieve a healthy, safe, and thriving region by building better systems and improving collaboration through regional partnerships and collective impact (8). The region is fortunate to have several longstanding binational partnerships that enable regular cross-border communication, coordination, and collaboration which have existed for decades.

These critical partnerships represent governmental agencies at the local, State and Federal levels on both sides of the border as well as with academic, medical, and non-profit organizations. Several HHSA departments work binationally and oversee the coordination of large binational initiatives. For instance, in collaboration with the US-México Border Health Commission, HHSA coordinates the annual binational *Love Your Heart/Ama Tu Corazón* campaign in all states along the US-Mexico border to raise awareness about heart health by offering free blood pressure screenings (9). The HHSA Office of Border Health has existed for 27 years with the role of facilitating communication and collaboration among the health agencies at the local level in San Diego and Tijuana (10). This is the only County-level border health office across the entire border region.

Another example includes the 15-year collaboration among the County of San Diego and Imperial County's Epidemiology Programs, the California Department of Public Health's Office of Binational Border Health—Border Infectious Disease Surveillance Program, and the State of Baja California on binational surveillance, case investigation, and reporting. This partnership is essential for prompt notification of new cases in border counties, coordinating binational case investigations, containment, and mitigation strategies.

When emergencies or situations requiring immediate response arise, such as with the COVID-19 pandemic, HHSA utilizes established communication pathways to communicate with its counterpart, ISESALUD Tijuana Health Services Jurisdiction (which also represents the jurisdictions of Tecate and Rosarito) and other key binational partners.

## Intervention Description

When the COVID-19 pandemic began in early 2020, regional public health leaders in San Diego, California and Tijuana, Baja California immediately began communicating and sharing data, updates, response strategies and offering support to one another. Due to existing and longstanding cross-border collaboration and partnerships, this effort unfolded quickly and seamlessly through traditional communication channels.

Simultaneously, the County of San Diego established a larger incident command structure to respond to the pandemic and to address coordination across disciplines. The County created nine sectors and 13 sub-sectors to facilitate regular communication, coordination, resource allocation, and address emerging needs. HHSA leaders who regularly work with Mexico, noted the urgency of developing a binational response strategy and the “Binational Sector” was formed with a local focus on the San Diego-Tijuana transborder region. The County of San Diego Board of Supervisors understood the importance of a dedicated binational response and recognized the transborder community in press conferences to create broad awareness and support.

The Binational Sector is led by the County Community Operations Officer within the Department of Homeless Solutions and Equitable Communities (HSEC) with support from a team comprised of HSEC and Public Health Services, Office of Border Health staff. The Binational Sector Lead and team regularly facilitate cross-border collaboration, outside of the pandemic, in multiple areas related to public health and emergency preparedness. The Binational Sector's main role is to share data, updates for situational awareness, response strategies, guidance, establish connections, facilitate equitable testing and vaccine access, and provide general coordination support to partners on both sides of the border.

## Partnerships

### ISESALUD Tijuana Health Services Jurisdiction

In partnership with ISESALUD Tijuana Health Services Jurisdiction, the Binational Sector developed infrastructure to formalize regular cross-border communication with key binational partners to address COVID-19 in the San Diego-Tijuana transborder region. They have remained an essential partner throughout the pandemic and on October 26, 2021, the County of San Diego Board of Supervisors recognized ISESALUD Tijuana Health Services Jurisdiction as a formal *Live Well San Diego* partner for their collaboration over two decades addressing mutual concerns in the areas of infectious and chronic disease prevention, health promotion, capacity building, and emergency preparedness, and many other important topics.

### Consulate General of Mexico in San Diego

The County of San Diego has worked closely with the Consulate General of Mexico (Mexican Consulate) in San Diego for decades on cross-border issues and the partnership has facilitated important initiatives throughout the pandemic. Not only have they played an essential role through advocacy and coordinating key activities, but the Latino community also trusts information from the Consulate and feels safe accessing their services at their offices in Central San Diego. Since the beginning of the pandemic, the County remained in close communication with the Consulate, sharing concerns, data, recommendations, and collaborating on outreach, testing, and vaccines efforts.

### US-México Border Health Commission

The US-México Border Health Commission is also a longstanding and critical partner on multiple binational endeavors. Outside the pandemic, for years they have served as an important leader on all aspects of binational health communication and collaboration. Throughout the pandemic, they have provided guidance, made critical connections, and ensured a broader conversation was taking place among all the border states in both the United States and Mexico to address challenges, share best practices, and identify improvement opportunities.

## Methods

The Binational Sector is comprised of three main functions: cross-border communication, testing and vaccine efforts, and binational coordination.

### Cross-Border Communication

To facilitate ongoing formal communication with binational partners, the Binational Sector established weekly video conference meetings in partnership with ISESALUD Tijuana Health Services Jurisdiction and other key partners, primarily representing the government, academic, and healthcare sectors. The video conference meetings have a local focus and each one includes an epidemiological update from both the County of San Diego and the ISESALUD Tijuana Health Services Jurisdiction, followed by a discussion among participants. To meet the needs of the bilingual audience, the Binational Sector contracts with a company that provides simultaneous English-Spanish interpretation *via* the Zoom platform. The Binational Sector also produces a biweekly, bilingual COV19 Binational newsletter highlighting epidemiological data at the local, state, and federal levels of the United States and Mexico, the latest local updates, and other helpful and timely resources for both California and Baja California.

Outside the scheduled video conference meetings, the Binational Sector plays a key role in facilitating regular communication among binational partners. The Binational Sector Lead also presents to various stakeholders on the COVID-19 transborder response as well as responds to numerous inquiries, varying in complexity, and confers with internal and external experts to provide responses and guidance. Further, with the goal of streamlining messaging and presenting consistent recommendations, the Binational Sector prioritized outreach and communication through media opportunities to reach the transborder community to increase confidence and adherence to public health measures.

### Testing and Vaccine Efforts

The County established a regional COVID-19 testing and vaccination ecosystem with health equity in the center. It was a priority to place accessible sites in disproportionately impacted communities, including many in the southern region of the County, where a significant proportion of the transborder population resides. This was achieved through working with trusted partners to provide testing and vaccines at their frequented locations. Partners included City leaders, the Mexican Consulate, churches, markets, and others who offered accessible sites throughout the region.

### Binational Coordination

In addition to the specific focus areas described previously, the Binational Sector provides and facilitates general support among many partners. Examples include facilitating multiple transborder Personal Protective Equipment donations, connecting partners with philanthropic opportunities, and troubleshooting unique challenges and requests.

## Results

### Cross-Border Communication

#### Video Conference Meetings

The Binational Sector began hosting video conference meetings on March 26, 2020, in partnership with ISESALUD Tijuana Health Services Jurisdiction and other key binational partners (primarily government agencies). The meetings focused on the San Diego-Tijuana transborder region. From March 2020 to April 2021, the meetings took place every 2 weeks. Beginning May 2021, the meetings moved to a monthly basis. At the time this article is submitted for publication, thirty-eight video conference meetings will have taken place.

At each meeting, the Binational Sector Lead facilitates and provides relevant updates related to binational specific efforts and the broader response. The County of San Diego Public Health Officer and the ISESALUD Tijuana Health Services leadership team (e.g., Director, Deputy Director, or Chief Epidemiologist) provide epidemiological summaries and related guidance. Following the epidemiological and general updates, a discussion takes place as well as an occasional presentation. Presentation examples include the Baja California Secretary of Health presenting their business safe reopening guidelines and the UN Refugee Agency in Tijuana sharing an overview of how the COVID-19 pandemic impacted Tijuana's migrant shelters.

#### Newsletter

Between April 2020 and December 2021, the Binational Sector produced and disseminated 35 newsletters to a binational audience. Each newsletter contained updated local, state, and federal epidemiological data and guidance from the United States and Mexico. As the pandemic evolved, it mirrored the needs of the moment such as updated testing and vaccine accessible locations for the transborder community. It also highlighted donation opportunities, and other helpful and timely resources related to mental health services, treatment opportunities, and other support services in both California and Baja California.

#### General Communication

The Binational Sector Lead and team respond to frequent complex inquiries and requests and coordinate with internal and external partners to develop guidance and responses. Due to the close relationships with binational partners, any concern that is communicated is flagged and elevated immediately for a response and guidance. Examples include data requests to illustrate the impact of the COVID-19 pandemic on Mexican-born nationals residing in San Diego and clarification questions related to vaccine eligibility in San Diego for the transborder population that reside in Mexico.

The Binational Sector Lead has also been invited to present on its activities on various panels, webinars, community presentations, and conferences. These opportunities have facilitated a broader conversation among local, state, national, and border state partners. There are many webinars and calls that take place on a regular basis to share information, exchange data and discuss effective strategies. One example in which the Binational Sector Lead participated in a panel discussion was during a *COVID-19 Virtual Seminar Along the United States Mexico Border Region*, hosted by the US-México Border Health Commission, in collaboration with the University of Arizona. The panelists shared COVID-19 data and examples of response best practices.

#### Media

At the time of this publication, the Binational Sector Lead has participated in several press events and more than 60 bilingual interviews on multiple TV and radio stations, in addition to print publications, that reach a transborder audience. As the pandemic has evolved, the topics and nature of the conversations have changed to reflect the priorities of the moment. At the beginning of the pandemic, topics centered around non-pharmaceutical interventions, promoting public assistance programs, addressing mental health, providing testing information, data, and addressing the disproportionate impact on the Latino population. As testing became more available in the southern part of the region, and the port of entry testing site was established, the interviews shifted focus to a conversation on the enhanced outreach campaign to the transborder Latino population and the use of promotores to help navigate online appointment platforms. Later interview topics centered on school reopening guidelines, the vaccine rollout strategy, and misinformation surrounding the vaccine.

The Binational Sector Lead has also participated in several press conferences with city and community partners on COVID-19 collaborative efforts and responded to numerous written media inquiries for local publications that reach a transborder audience.

### Testing and Vaccine Efforts

#### Testing Sites

In the Fall of 2020, with the support of the County of San Diego Board of Supervisors, the HHSA Director, and other key community partners, the Binational Sector Lead facilitated the opening of strategic testing sites at various locations (schools, markets, churches, etc.) frequented by the transborder Latino population. One key location was the Mexican Consulate and testing events took place at their site weekly between September 14, 2020 and June 28, 2021 and during popup events until February 3, 2022. In total, there were 39 testing days and 8, 029 tests performed in partnership with the County of San Diego. To further increase access and trust, promotores assisted individuals in booking testing appointments. The Consulate testing site was so successful that it later transitioned to becoming a vaccination site.

Another strategic testing site was implemented at the San Ysidro Port of Entry to create quick and easy access for individuals who cross the border daily, many of whom comprise San Diego's essential work force. Customs and Border Protection played an important role in approving access, providing logistical support, and streamlining the population flow. The County of San Diego provided nursing staff to assist with administering the tests. The San Ysidro Port of Entry testing site was open every weekday for 217 days and performed 22, 874 tests from August 12, 2020 to June 30, 2021.

#### Vaccine Sites and Events

The Mexican Consulate, UC San Diego, the Secretary of Health in Baja California, U.S. Customs and Border Protection, and many binational business partners, started a pilot border vaccine project in May 2021. Over a 2-week period, nearly 10,000 vaccines were administered to US-owned Tijuana maquiladora employees at the San Ysidro Point of Entry. The pilot was so successful that it was extended to eventually vaccinate over 27,000 people and was replicated in other jurisdictions along the US-Mexico border.

The Binational Sector Lead also played a role, along with the County of San Diego's “T3” team (T3 - a strategy that focuses on testing, tracing, and treatment), the California Department of Public Health, and the US Consulate in Tijuana, to provide COVID-19 vaccines to H-2A workers crossing into the U.S. from Tijuana, Mexico, at vaccination sites near the US-México border. The CDC supported the project through promotion at local agricultural sites.

The Mexican Consulate eventually became a regular vaccine site for mobile, popup and special vaccine events. In total, there were 41 vaccine events and 3, 256 vaccines administered in partnership with the County of San Diego between March 24, 2021 and December 2, 2021. Another strategic vaccine site was at the Southwestern College located less than ten miles from the San Ysidro and Otay Mesa ports of entry. The site was open for 79 days and 18, 866 vaccines were administered in partnership with the County of San Diego between February 2, 2021 and June 29, 2021.

Two other Binational Sector-supported vaccine events focused on children ages 12–18 years old from Baja California. One took place in San Diego at the Mexican Consulate on November 5, 2021 and the other took place on November 18, 2021 at the centrally-located Tijuana Cultural Center in Tijuana, Baja California. It was a collaboration with the Baja California Secretary of Health, ISESALUD Tijuana Health Services Jurisdiction, the Mexican Consulate in San Diego, the County of San Diego, the US-México Border Health Commission, Tijuana Innovadora, and many other organizations. Vaccines were donated by the County of San Diego HHSA.

### Binational Coordination

#### Personal Protective Equipment Donations

The Binational Sector provides general support with coordinating transborder collaborative projects. It has coordinated several cross-border Persona Protective Equipment (PPE) donations over the last 18 months. One substantial request involved the Binational Sector's coordination of a large State of California PPE donation *via* the County of San Diego to the Baja California ISESALUD Tijuana Health Services Jurisdiction who disseminated items to public hospitals, fever clinics, Tijuana Red Cross and Tijuana Civil Protection. This request involved coordinating with local and state officials in California as well as the Mexican Consulate, the Baja California Secretary of Health, and the revenue service of the Mexican federal government. After a few months of communication, permit processing and inventory accounting, the request was approved by the Mexican federal government and on November 12, 2020, 83 pallets of PPE were transported by four semi-trucks from a warehouse in San Diego to a warehouse in Tijuana. The same day, the Baja California Secretary of Health and ISESALUD Tijuana Health Services Jurisdiction arranged a reception and press conference with key partners including the Binational Sector Lead and Mexican Consulate to acknowledge their appreciation of the large donation and overall partnership.

#### Partner Coordination

Another important role the Binational Sector has played throughout the pandemic relates to aligning interests and bridging key partners and stakeholders. The Binational Sector facilitated communication among multiple partners interested in volunteering, donating, addressing complex challenges.

One example in which the Binational Sector provided coordination was when the need was identified for streamlining a notification pathway for individuals with COVID-19 who are released from hospitals in San Diego County and returning to their home in Mexico. Ensuring that each entity in the pathway is given advanced notification, is essential for preparedness, to take the necessary precautions and to ensure the patient is supported along their journey and when they reach their destination. The Binational Sector facilitated meetings among the County of San Diego Emergency Medical Services, the County of San Diego's Epidemiology program, the California Department of Public Health, Office of Binational Border Health, Centers for Disease Control and Prevention Division of Global Migration and Quarantine US-Mexico Unit, Tijuana Red Cross, and the ISESALUD Tijuana Health Services Jurisdiction to develop a notification pathway for Mexico-bound discharged patients who are traveling *via* ambulance. Once all participating entities provided input and agreed on a process, the pathway was finalized and implemented across the San Diego County hospital system.

## Discussion

Recognizing the region's interconnectedness, that San Diego County and Tijuana share a transborder population, and the fact that disease spread is multi-directional, the need for a Binational Sector to coordinate cross-border communication, collaboration, and coordination was essential. Fortunately, the County of San Diego HHSA and its counterpart ISESALUD Tijuana Health Services Jurisdiction had a strong existing relationship and had collaborated on multiple endeavors for decades prior to the COVID-19 pandemic. The pandemic deepened this relationship due to the increased communication and coordination between the two entities, further solidifying their commitment to work together and support one another. This partnership underscores the need to maintain and foster the broader collaborative transborder agency network.

The pandemic has highlighted the need to further understand the unique life circumstances of transborder communities and how they impact health outcomes. A significant number of daily border crossings continued throughout the pandemic, even with the Department of Homeland Security's border crossing restrictions in place, given the essential nature of cross border activity in the region. It is vital to take this into consideration when designing binational public health interventions. There is a need to continue to identify opportunities to intervene, support, and connect the transborder community to available resources. Further, it is important to examine ways to streamline public health messaging to ensure the guidance is consistent on both sides of the border, especially during public health emergencies or natural disasters.

The COVID-19 pandemic has also highlighted inequities that exist in our communities of color and how they contribute to health disparities. One of the greatest challenges has been implementing health measures and prevention strategies while recognizing that these changes greatly impact the economy and when the economy suffers, the already disadvantaged communities suffer even more. One key community is the transborder essential work force. To mitigate these challenges, the Binational Sector focused on increasing access to testing and vaccine sites and conducting community outreach by employing promotores and partnering with media that reach a broad transborder audience.

There has also been a keen awareness of the mental health impact of the pandemic on the broader community and one can imagine the impact has been even greater on the transborder Latino community. To address these types of inequities and health disparities, the San Diego County HHSA inaugurated a new department, the Department of Homeless Solutions and Equitable Communities, to ensure that all operations are conducted through an equity lens. This new department will have a prominent binational focus within its Office of Immigrant and Refugee Affairs.

The COVID-19 pandemic has also provided an opportunity to strengthen emergency preparedness and cross-border response activities. Several partners including the City of San Diego, the San Diego County Office of Emergency Services and Emergency Medical Services, the California Governor's Office of Emergency Services, and Civil Protection and fire departments in Baja California have a history working together and they worked closely throughout the COVID-19 pandemic. These relationships have deepened and have leveraged an opportunity to improve cross-border emergency communication infrastructure, protocols, and other processes.

Other opportunities for improvement include dedicating resources to support a robust infrastructure to facilitate binational contact tracing and case notification, additional testing, treatment, and vaccine sites at ports of entry, and streamlining processes for sharing and donating resources across the border.

## Conclusion

The interdependence between the two sister states creates a need for mutual support during times of crisis. From a public health perspective, this region is considered one with a shared community and shared health challenges. With the constant fluidity, diseases can spread quickly in both directions, as is proving evident with the COVID-19 pandemic. For these reasons, it is critical for leaders on both sides of the border to communicate regularly and work together to support the transborder community. It was essential to implement a formal structure to facilitate regular binational communication, coordination, and collaboration to respond to COVID-19 jointly and effectively. Coming together during this challenging time has only strengthened the existing partnerships and cross-border network, bringing various agencies and entities together in a more profound way. By creating an effective infrastructure and deepening cross-border relationships, it will be possible to reestablish this sector in the event of any future public health emergency or natural disaster and it will be feasible to carry out the same successful and impactful operations.

Prior to the pandemic and ongoing are several other cross-border collaborative efforts at the local, state, and federal levels across the border region. These efforts include academic, government, and non-profit partners and have focused on volunteer efforts, philanthropy, testing, vaccines, and coordinated medical care. At the time of this publication, the County of San Diego Binational COVID-19 Sector is the only county-level formal cross-border structure created in response to the pandemic. It received a 2021 National Association of County and City Health Officials Innovative Practice Award for “Ongoing cross-border (San Diego-Tijuana) COVID-19 collaboration” and a National Association of Counties 2022 Achievement Award for “COVID-19 Cross-Border Vaccination Efforts.” Due to its logical structure and components, it can be replicated in other global cross-border regions.

## Data Availability Statement

The original contributions presented in the study are included in the article/supplementary material, further inquiries can be directed to the corresponding author/s.

## Author Contributions

BJ and JK drafted all content for the article. Both authors contributed to the article and approved the submitted version.

## Conflict of Interest

The authors declare that the research was conducted in the absence of any commercial or financial relationships that could be construed as a potential conflict of interest.

## Publisher's Note

All claims expressed in this article are solely those of the authors and do not necessarily represent those of their affiliated organizations, or those of the publisher, the editors and the reviewers. Any product that may be evaluated in this article, or claim that may be made by its manufacturer, is not guaranteed or endorsed by the publisher.
